# Drug administration errors in Latin America: A systematic review

**DOI:** 10.1371/journal.pone.0272123

**Published:** 2022-08-04

**Authors:** Lindemberg Assunção-Costa, Ivellise Costa de Sousa, Maria Rafaela Alves de Oliveira, Charleston Ribeiro Pinto, Juliana Ferreira Fernandes Machado, Cleidenete Gomes Valli, Luís Eugênio Portela Fernandes de Souza

**Affiliations:** 1 Department of Medicine, School of Pharmacy, Federal University of Bahia, Salvador, Bahia, Brazil; 2 Department of Pharmacy, University Hospital Professor Edgard Santos, Salvador, Bahia, Brazil; 3 National Institute for Pharmaceutical Assistance and Pharmacoeconomics, Salvador, Bahia, Brazil; 4 Health Department of the State of Bahia, Salvador, Bahia, Brazil; 5 Institute of Collective Health, Federal University of Bahia, Salvador, Bahia, Brazil; Public Library of Science, UNITED KINGDOM

## Abstract

**Purpose:**

This study systematically reviewed studies to determine the frequency and nature of medication administration errors in Latin American hospitals.

**Summary:**

We systematically searched the medical literature of seven electronic databases to identify studies on medication administration errors in Latin American hospitals using the direct observation method. Studies published in English, Spanish, or Portuguese between 1946 and March 2021 were included. A total of 10 studies conducted at 22 hospitals were included in the review. Nursing professionals were the most frequently observed during medication administration and were observers in four of the ten included studies. Total number of error opportunities was used as a parameter to calculate error rates. The administration error rate had a median of 32% (interquartile range 16%–35.8%) with high variability in the described frequencies (9%–64%). Excluding time errors, the median error rate was 9.7% (interquartile range 7.4%–29.5%). Four different definitions of medication errors were used in these studies. The most frequently observed errors were time, dose, and omission. Only four studies described the therapeutic classes or groups involved in the errors, with systemic anti-infectives being the most reported. None of the studies assessed the severity or outcome of the errors. The assessment of the overall risk bias revealed that one study had low risk, three had moderate risk, and three had high risk. In the assessment of the exploratory, observational, and before-after studies, two were classified as having fair quality and one as having poor quality.

**Conclusion:**

The administration error rate in Latin America was high, even when time errors were excluded. The variation observed in the frequencies can be explained by the different contexts in which the study was conducted. Future research using direct observation techniques is necessary to more accurately estimate the nature and severity of medication administration errors.

## 1. Introduction

On average, 10% of patients admitted to hospitals suffer from some type of adverse event related to medications, half of which are preventable [1]. Recently, concerned with this scenario, the World Health Organization launched the third patient safety global challenge to reduce medication use harm by 50% in five years [2]. Harm to patients attributed to medication errors (ME) and preventable adverse events are among the most common hospital incidents. They have significant clinical, economic, and social consequences [3]. The global economic impact of medication error is approximately US$ 42 billion annually [4], which is 0.7% of the global total health expenditure. However, much of the evidence on medication errors is derived from developed countries [4].

Research carried out in developing countries revealed that 2.5%–18.4% of hospital admissions were associated with adverse events, of which 84% were preventable and 30% resulted in the death of the patient [5]. These rates were higher than those identified in developed countries, probably because of the low qualifications of health professionals and inadequate infrastructure of health systems [6].Understanding the context and solutions for reducing the risks of drug-related harm in developing countries is essential for providing safe and effective care to the population [6].

Medication errors can be understood as those arising during prescription, dispensing, and administration of medications [7]. Several studies have shown high frequency of medication errors [8–12]. Some recent systematic reviews using direct observation alone have shown mean medication administration error (MAE) rates of 8–10% (excluding time errors) [12–15]. The detection and quantification of medication administration errors are essential to establish the frequency at which they occur and identify underlying causes and factors that allow interventions to reduce their occurrence [14].

Administration is the final stage of the drug use process, and errors in this stage are least likely to be intercepted before reaching the patient [16]. Medication administration error is defined as any discrepancy between the prescribed drugs and the drugs administered to the patient [14,17]. Medication preparation errors at the ward level are also considered as administration errors. Prescription and dispensing errors are excluded from this review.

Several methods are used to measure medication administration errors, including self-reporting, incident reporting, medical record review, trigger tool, and direct observation. Each has its own advantages and disadvantages. Incident reporting and self-reporting methods produce error rates that underestimate the prevalence of errors in medication administration. Direct observation is the most appropriate method for accurately identifying a variety and significant number of medication administration errors. This allows the comparison of medication administration error rates among published studies. A disadvantage of this method is that it is more labor intensive and expensive and can lead to changes in the participants’ behavior in the observers’ presence [14].

Most systematic reviews of medication administration errors in hospitals are published in English and include very few studies conducted in Latin America because they exclude studies in Portuguese and Spanish [13,14,18]. No systematic review has reported the incidence of medication administration errors based on the direct observation method in Latin America [19]. Two reviews found in the literature, published in Portuguese and Spanish, studied nurses in Latin American hospitals and evaluated errors in the preparation and administration of medications. One study attempted to describe the qualitative aspects [20,21], while the other sought to identify the types and factors associated with them [21]. Therefore, to the best of our knowledge, this is the first systematic review that aims to determine the frequency and nature of medication administration errors identified through the direct observation method in Latin American hospitals.

## 2. Methods

This systematic literature review was conducted in accordance with the Preferred Reporting Items for Systematic Reviews and Meta-Analysis (PRISMA) 2020 guidelines [22,23].

### 2.1 Eligibility criteria

We included studies reporting the rate of administration errors using only the direct observation method, published between 1946 and March 2021 in Portuguese, English, or Spanish, performed in hospitals in Latin American countries (Argentina, Bolivia, Brazil, Chile, Colombia, Costa Rica, Cuba, El Salvador, Ecuador, French Guiana, Guatemala, Haiti, Honduras, Mexico, Nicaragua, Panama, Paraguay, Peru, Puerto Rico, Dominican Republic, Uruguay, or Venezuela). Prospective, cross-sectional, observational, or interventional before-after studies were included in our analysis. For interventional before-after studies, only the administration error rate calculated in the period before the intervention was considered.

We excluded studies such as narrative reviews; guides; protocols; qualitative studies; case reports; studies that used interviews, questionnaires, or focus groups to identify factors or causes of medication errors or professionals’ feelings regarding medication errors; studies that did not stratify the types of medication errors; conference summaries that did not provide enough information to determine the prevalence and nature of medication administration errors; studies on medication administration errors associated with a medication or medication class or that reported only a subcategory of administration errors (e.g., dose error); studies that assessed the rate of administration errors during home care; and studies that provided only information about serious medication administration errors, instead of information about all medication errors.

### 2.2 Information sources

Two researchers (MR and IC) independently reviewed the following seven electronic databases: LILACS via Bireme, PubMed, SciELO, Scopus, Latindex, Embase, and CINAHL, applying search strategies described in S1 appendix. Gray literature (searched using Google Scholar), reference lists of included studies, and relevant review articles were manually searched to identify additional eligible studies. Unpublished papers obtained from the thesis and research database files from academic libraries were also reviewed. The search was conducted between August 2019 and March 2021.

### 2.3 Search strategies

Search strategies aimed to retrieve studies on medication errors, especially administration errors in hospital care, carried out in Latin American countries, as in the example: (“medication error$” OR “administration error$” OR “medication preparation” OR “omission error$” OR “medication handling”) AND “hospital$” AND (“Latin America” OR “Argentina” OR “Bolivia” OR “Brazil” OR “Chile” OR “Colombia” OR “Costa Rica” OR “Cuba” OR “El Salvador” OR “Ecuador” OR “Guatemala” OR “Haiti” OR “Honduras” OR “Mexico” OR “Nicaragua” OR “Panama” OR “Paraguay” OR “Peru” OR “Puerto Rico” OR “Dominican Republic” OR “Uruguay” OR “Venezuela”).

We also reviewed the gray literature, reference lists of the included studies, and relevant reviews to minimize the risk of loss of eligible studies.

### 2.4 Selection process

Eligibility was initially assessed by reading the title and abstract of each article. When the title and abstract did not provide sufficient information to determine whether the study met this review’s objectives, the paper was retrieved and thoroughly read to analyze its fit with the inclusion and exclusion criteria. All eligible studies were retrieved for full-text reading. Two independent reviewers (MR and CI) applied the eligibility criteria, and the results were subsequently validated by a third reviewer (LAC) to consolidate the final selection of studies. Discrepancies were resolved by consensus among the three reviewers after discussion.

### 2.5 Data collection process

Data extraction was performed independently and in pairs. We developed a standardized form on a Microsoft Excel® spreadsheet (version 16.43, Mac) to extract the authors’ names, year of publication, country of origin, hospital where the study was conducted, study duration, study type, data collection method (who the observer was, the number of observers, and the observed professional), the numerator (administration errors observed or recorded), the denominator (type and value), the definition of medication error or medication administration error, disguised and undisguised observation technique, type of errors (omission, dose, and time) based on the classifications proposed by ASHP [24], NCC MERP [25], or Barker and Allan [17] (S1 Annex), the severity of medication error or medication administration error and which classification was used, administration route, risk factors, therapeutic classes involved with medication administration error, and the frequency of administration errors observed or recorded. IC and MR extracted the data independently, and the results were validated by a third reviewer (LAC). Discrepancies were resolved by consensus among the reviewers.

Some authors were contacted to clarify doubts about the findings of the studies, especially regarding error-frequency calculations.

### 2.6 Evaluated outcomes

We extracted the following data from each study:

1. Study characteristics: country, year, duration, design, and clinical setting;
2. Identification of MAE: definition of MAE, observation method, frequency of administration errors, and severity assessment of MAE;
3. Information relating to the MAE: frequently reported medications; medication errors involving intravenous administration route, and drugs associated with medication errors.

### 2.7 Risk of bias assessment

We used the Joanna Briggs Institute (JBI) checklist for analytical cross-sectional studies for each cross-sectional study included. The tool comprises eight questions to determine the quality of studies [26]. At the end of the assessment, according to the criteria met by each study, we considered high risk of bias as studies that met 0% to 50% of the criteria, moderate risk of bias as those that met 51% to 75% of the criteria, and low risk of bias as those that met 76% to 100% of the criteria.

For observational, multicenter, exploratory, and interventional before-after designs, we applied the Newcastle-Ottawa Quality Assessment Form for Cohort Studies. This tool is structured into three domains of bias (selection, comparability, and outcome) that include questions that inform the risk of bias judgments. Based on the obtained scores, studies were classified as having good, fair, or poor quality [27].

### 2.8 Effect measures

The denominator extracted from the studies was the “Total Opportunity of Error” (TOE), defined as the total number of doses administered, correctly or incorrectly, plus the number of doses omitted. Whenever possible, we converted the values presented in the studies into TOE. The numerator data represent the total number of errors observed. When the studies evaluated the impact of an intervention using the before-after method, we extracted only the data from the pre-intervention period. The total ME rate was used for multicenter studies.

The studies included in this review showed a wide variation, and for this reason, the median error rates were calculated with interquartile intervals (IQR). Median error rates were calculated with and without time error rates. For studies that reported different error rates for the medication administration and preparation stages in inpatient units, the combined data were used to build a total administration error rate. The error rate was used in the pre-intervention stage in “before-after” intervention studies.

### 2.9 Synthesis methods

Meta-analysis was not performed due to methodological differences among studies, within-study biases, and diversity of outcomes. Instead, we presented the results of individual studies in descriptive tables, according to the identified medication administration error frequency.

## 3. Results

**Fig 1:**
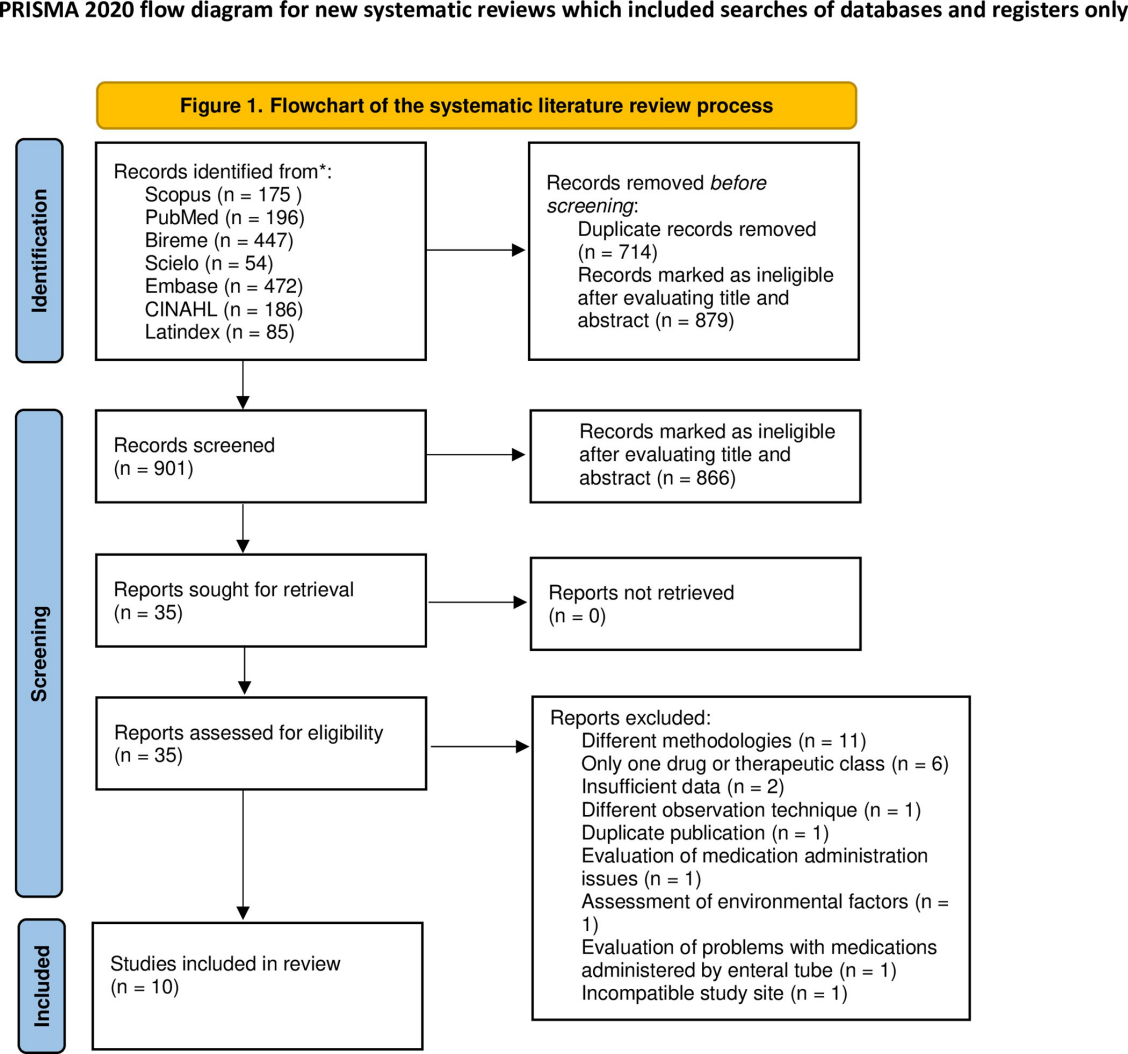
Flowchart of the systematic literature review process. Reference: Page MJ, McKenzie JE, Bossuyt PM, Boutron I, Hoffmann TC, Mulrow CD, et al. PRISMA 2020 statement: An updated guideline for reporting systematic reviews. BMJ 2021;372:n71. doi: 10.1136/bmj.n71. For more information, visit: http://www.prisma-statement.org/.

The search for information sources resulted in the initial identification of 1,615 papers, of which 914 duplicates were excluded. Another 666 papers were excluded after reading the titles and abstracts because they did not meet the inclusion criteria. The remaining 35 papers were retrieved for full-text reading and detailed analysis. Finally, ten papers were included in this review (Fig 1).

### 3.1 Characteristics of included studies

#### 3.1.1 Country and year of publication

Eight (80%) of the included studies were conducted in Brazil and two (20%) in Chile and were published between 2006 and 2018. Detailed information is provided in Table 1.

**Table 1:** Characteristics of the included studies.

Reference	Country of origin	Hospital context	Duration (days)	Study type	Observation method	Participants		Denominator, n	Numerator	Frequency	Error definition used	Error type*
						Observer, n	Observed, n					
Costa et al., 2006 [28]	Brazil	2 units (1 MC and 1 SC) of 1 private hospital and 1 unit (MC) of 1 public hospital	30	Cross-sectional	DO	NI, 2	NI	TOE, 638	209	32.9%	Barker et al., 2002	Omission (10.5%) Non-prescribed dose (10.2%) Time error (8.3%) Wrong dose (3.3%)
Opitz, 2006 [29]	Brazil	1 unit (MC) of 1 teaching hospital	15	Observational and cross-sectional	DDO	Nurses and nursing students, NI	Nurses (3), Nursing Assistant (2), and Nursing Technician (17), 22	TOE, 1129	404	35.8%	NCC MERP	Time error (19.0%) Omission error (9.4%) Dose error (5.7%) Unauthorized medications (1.4%)
Anselmi et al., 2007 [30]	Brazil	5 units (IM, pediatrics, obstetrics, SC, and emergency) of 3 hospitals	35	Cross-sectional	DO	Nurses (3) and nursing students (14), 17	Nurses (49), Nursing Assistants (44), and Nursing Technicians (27), 120	TOE, 1315	104	16%	Barker et al., 2002	Wrong dose (9.2%) Dose omission (5.3%) Wrong patient (1.2%) Wrong medication (0.3%)
Reis et al., 2009 [31]	Brazil	5 units (MC) of 5 teaching hospitals	30	Multicenter exploratory/ descriptive	DO	NI, 15	Nursing professionals, NI	TOE, 4958	1500	30.3%	Barker et al., 2002	Time error (77.3%) Wrong dose (14.4%) Route error (6.1%) Unauthorized medication (1.7%)
De Bortoli Cassiani et al., 2010 [32]	Brazil	6 units of MC of 6 hospitals, 4 of which were teaching hospitals	30	Cross-sectional	DDO	Nurses (6) category NI (18), 24	Nursing professionals, NI	TOE, 6169	1049	17%	NCC MERP	Time error (53.8%) Wrong dose (26.4%) Unauthorized medications (9.8%) Wrong route (8.5%)
Teixeira and Cassiani, 2010 [33]	Brazil	1 unit (MC) of 1 university hospital	30	Cross-sectional	DO	NI	Nursing Assistants, Nursing Technicians, NI	TOE, 824	74	9%	NCC MERP/ ASHP	Dose errors (24.3%) Time errors (22.9%) Unauthorized medications (13.5%) Technique errors (12.2%)
Romero et al., 2013 [34]	Chile	2 SC of 1 teaching hospital	180	Before/After	DDO	Pharmacists, NI	Nursing Teams, NI	TOE, 194	66	34%	Ferner & Aronson	Administration error (26%) prescription error (10%) Preparation error (7%) Transcription error (4%)
Grou Volpe, 2014 [35]	Brazil	1 unit (MC) of 1 general hospital	10	Cross-sectional	DO	Nurses, 2	Nurses (8) and Nursing Technicians (16), 24	TOE, 531	337	64%	NCC MERP	Time errors (48.5%) Dose omissions (9.5%) Wrong dose (1.7%) Monitoring errors (0.4%)
Smith M, 2014 [36]	Chile	1 ICU of 1 university hospital	180	Observational	DDO	Pharmacists and pharmacy students, NI	NI	TOE, 132	52	38.6%	NCC MERP	Time error (76.8%) Incomplete prescription (13.8%) Dispensation error (7%)
Mendes et al., 2018 [37]	Brazil	1 FA of 1 university hospital	180	Cross-sectional	NDDO	NI, 1	Nursing Assistants, Nursing Technicians, and Nurses, 303	TOE, 303	33	10.8%	NCC MERP	Time error (5.6%) Dose error (2.6%) Technique error (2.6%)

MC: Medical clinic, SC: Surgical clinic, IM: Internal medicine, ICU: Intensive care unit, FA: First aid, DO: Direct observation, DDO: Disguised direct observation, NDDO: Non-disguised direct observation, NI: Not informed.

*Four types of errors described most frequently in each included study.

#### 3.1.2 Study locations

The studies were conducted in 22 hospitals, of which 14 (64%) were university or teaching hospitals and 8 (36%) were general hospitals. The units chosen for observation were medical clinic units (16, 61.5%), surgical clinics (4, 15.4%), emergency care (2, 7.7%), intensive care (1, 3.8%), pediatrics (1, 3.8%), obstetrics (1, 3.8%), and internal medicine (1, 3.8%). Four (40%) studies were conducted in two or more institutions [28,30–32]. The drug distribution systems found in these hospitals were individualized [28], mixed [28,29], and unit-dose [34]. Other studies did not report the distribution system used.

#### 3.1.3 Study design

Seven (70%) cross-sectional studies, two (20%) “before and after” studies, and one (10%) descriptive, exploratory, multicenter study were included. Disguised direct observation was performed in four (40%) studies to assess medication administration, a method in which the observed team is not aware of the study to avoid changes in usual behavior. The individual professional category observed was described in eight (80%) studies, represented by nursing professionals (nurses, nursing assistants, and technicians).

#### 3.1.4 Patient profile

The studies did not inform the age groups of the patients. However, patients from adult and pediatric units were included in the observations. Most of the observations were made in clinical units of hospitals (16, 61.5%), characterized in the studies as units providing care to patients with chronic diseases, using a high number of medications.

#### 3.1.5 Administration route

Two studies examined medication administration errors associated with intravenous drugs [30,37]. In one study, observations were restricted to doses administered either parenterally or enterally. The same study excluded from its evaluation medications administered by inhalation or through a continuous infusion pump [29]. Other studies evaluated errors that occurred without restrictions regarding the medication administration route.

#### 3.1.6 Observers and error detection

Nurses were the most frequent observers in the studies and were involved in data collection in four of the ten studies included; in one of them they were the sole responsible professional. Nursing students participated in the collection of three studies, pharmacists in two, and pharmacy students in one. The observer’s professional category was not described in four of the studies. Six studies (60%) confirmed the error when comparing the observations, registered in a specific form, to the medical prescription after the observation period [28,31–35]. Two studies (20%) confirmed the error simultaneously with the observation [29,30]. Two studies did not report whether the error was confirmed during or after the observation [36,37].

Six studies reported the training provided to the observer [29–32,34,36]. As described by Barker et al., proper training and technique are an important part of reducing bias or the Hawthorne effect in persons administering medication [38].

The contents addressed in this study included the concept of medication errors, types of errors, ways of approaching the person being observed, presentation, orientation, and discussion of the research instrument, culture of safety, medication use system, and detection and classification of. Three studies revealed a total training time of 20 hours [29,31,32].

#### 3.1.7 Error validation

Only one study [37] did not include two or more observers in data collection. Four studies (44.4%) among those with two or more observers reported that they underwent training to standardize the validation process [28,30,32,36]. Five studies validated the form used in data collection before the observation’s onset [29,31,32,34,36]. Three of them described that the validation was performed by experts on the subject [29,31,32]. Divergences were resolved by consensus among observers [30] or involving a supervisor [30]. One study reported that patient safety experts validated the data collected [31], and another reported the use of an external supervisor who collected the data from 10% of the observed patients and compared it with the observations of the other collectors [34]. Eight of the included studies stated that observers were instructed to intervene in errors that could harm patients. None of the studies evaluated the severity of errors.

#### 3.1.8 Error definition

Four different error definitions were used in the studies. The most frequently employed were NCC MERP [24] (6; 60%) and Barker [25] (3; 30%). One study adopted two definitions (ASHP [23] and NCC MERP [24]). One study used the definition of Ferner and Aronson [39].

### 3.2 Frequency of administration errors

#### 3.2.1 Denominator and numerator

All studies presented a denominator using the TOE definition. The numerator corresponded to the total number of errors observed during the data-collection period. The median error rates were 32% (IQR: 16–35.8%) and 9.7% (IQR: 7.4%–29.5%) without time errors.

#### 3.2.2 Frequently reported types of administration errors

The most frequent error was the wrong time error, defined as medication administration before or after one hour of the prescribed time [28–32] or drug administration before or after 30 minutes of the prescribed time [34,35,37]. The reported frequency of incorrect time errors in these studies ranged from 8.3% to 77.3%. Wrong dose errors were observed, with frequencies ranging from 1.7% to 26.4%. Omission errors were another common error subtype, with frequencies ranging from 5.3% to 10.5%.

#### 3.2.3 Intravenous administration route

Two studies investigated medication errors involving only drugs administered intravenously [30,37]. The most frequently described underlying errors were dose, omission, and incorrect time errors. Regarding medication preparation, the errors described were dose errors, lack of hand hygiene before preparation, non-use of aseptic techniques in preparation, incorrect identification of the medication, non-verification of the patient’s identification, and dilution of the medication in a volume below the manufacturer’s recommendation. The errors described in the administration stage were omission of medication, non-hand hygiene before administration, non-use of aseptic techniques for administration, and incorrect administration speed. One study [35] performed an analysis of the observed medication errors and the administration route, with 49.7% of the observed errors involving the intravenous route, 68% involving the administration, 56% involving preparation errors, and 44.4% involving wrong time errors. The study did not identify a statistical difference when considering the intravenous administration route as a risk factor for medication administration errors, as was the case for the other evaluated routes. Other included studies described the main types of errors observed, as described in Table 1.

#### 3.2.4 Drugs associated with medication administration errors

Four studies reported the classes [36] or therapeutic groups [31,34,35] associated with the observed medication administration errors according to the Anatomical Therapeutic Chemical code. The groups most frequently involved in medication administration errors were anti-infectives for systemic use, nervous system, blood and forming organs, cardiovascular system, digestive system, metabolism, and the respiratory system. One study [31] reported the frequency of medication administration errors associated with high-alert medications and a narrow therapeutic index. High-alert medications, most often involved in errors, were heparin, tramadol, and insulin. High-alert medications bear a heightened risk of causing significant patient harm when used in error [40].

The drugs with narrow therapeutic indices mentioned in the studies were heparin, vancomycin, and clindamycin.

### 3.3 Study quality evaluation

In the overall bias risk judgement for cross-sectional studies using the JBI assessment, one study was classified as having low risk, three as having moderate risk, and three as having high risk. In the analysis of the remaining studies using the Newcastle-Ottawa Quality Assessment, two studies were classified as having fair quality and one study as having poor quality (S3 Appendix).

## 4. Discussion

The median medication administration error rate was 32% (IQR: 16%–35.8%), with significant variability in the described frequencies (between 9% and 64%). When excluding time errors, the administration error rate ranged from 6.9% to 32.7% with a median of 9.7% and interquartile interval of 7.4% and 29.55%. The wide variation observed in frequencies can be explained by the different contexts in which the research was conducted, involving different types of hospitals, medication distribution systems, and professional categories, including students participating in data collection.

These studies adopted different classifications of medication errors. Barker [25] and NCC MERP [24] were the most frequent, whereas ASHP’s classification [23] was used in only one publication. Consequently, the error definitions varied in different studies. Only four studies reported observer training to ensure homogeneity in the identification of errors. The identified medication administration error rate was higher than that described in other systematic reviews [13,14]. However, it approached when time errors were excluded, with a median TOE of 9.7%.

The error rates identified in studies that evaluated only intravenously administered drugs were 10.8% and 16% [30,37]. One study [35] did not identify an increased risk of errors in the intravenous administration of drugs. These results differ from those of international systematic reviews that show a greater risk of errors (53.3%) in this route of medication administration [14].

However, the intravenous route was not identified as a risk factor for medication administration errors in other publications in the literature [41,42]. The intravenous administration route is associated with considerable complexity and more significant risks to the patient because intravenous drugs may require elaborate preparation and administration processes, leading to additional error opportunities compared with other routes [38]. One study did not include an aseptic technique in the preparation and administration of the observed errors [30].

The denominator “Total Opportunity of Error” was used in all the included studies, corroborating the literature that suggests TOE as the measure most frequently used for studies to identify medication administration errors based on direct observation [16,25]. As an observation technique, variations were identified in describing the data collection method used in each study: undisguised or disguised direct observation. The observer’s presence can lead the observed professional to be more careful or prone to error. However, the literature describes that participants tend to resume regular habits in their routine over time if the observer is discrete [14,43–45]. Adequate observer training can minimize the effects of observer presence [16,45].

The underlying error type most frequently described in eight of the ten included studies was wrong time error, similar to that observed by other authors [13,14]. The classification varied between studies, which considered 30 or 60 minutes as the time between the established time and the time when the medication was administered to determine the error. The relevance of this type of error is discussed in the literature, as they are usually classified as minor clinical errors. The clinical impact of incorrect time errors should be evaluated when timing is a critical factor in avoiding potential harm to patients [13,14].

After incorrect time errors, dose (wrong dose or non-prescribed dose) and omission errors were the most frequently described medication administration errors. Dose, time, and omission errors were frequent among studies that evaluated errors involving intravenous drugs [30,37]. These results were similar to those reported in the literature. One study [37] included aseptic techniques and non-hand hygiene among errors in the administration and preparation stages, which were not described in other studies [30,35] In the preparation stage, inadequate infusion rate and non-use of the aseptic technique were the most described errors, while incorrect dose and non-use of aseptic technique were the most common in the administration stage. Other published studies have included inadequate preparation techniques among the types of medication errors, which can result in a higher frequency of preparation errors [46,47].

The studies did not categorize the clinical relevance or severity of the error outcomes. Only four studies assessed the frequency of different therapeutic groups involved in medication administration errors. The profile identified was similar to that described in previous studies [14,48], with anti-infectious groups for systemic use, nervous system, blood and forming organs, cardiovascular system, respiratory and digestive systems, and metabolism as the most frequently involved in medication administration errors [14,48]. One study identified high-alert medications and those with a narrow therapeutic index as the most frequently described. The frequent description in the literature of these therapeutic groups as the ones most involved in medication errors highlights the need for attention owing to the high risk of medication administration error damage, especially those involving high-alert medications. It is necessary to establish strong barriers to prevent these errors. The efficacy of many drugs in the aforementioned therapeutic groups is associated with specific administration times, and it is essential to adopt strategies to reduce time errors [14,15,48].

To the best of our knowledge, this is the first systematic review on the prevalence and nature of medication administration errors in Latin American hospitals [19–21]. Owing to the scarcity of published information on medication administration errors in Latin American countries, this review aimed to include only studies conducted in Latin American hospitals. This study had some limitations. First, only two countries, Brazil and Chile, have reported studies using direct observation techniques to identify medication administration errors, which may not represent the rate in other Latin American countries. Another critical factor was the heterogeneity of the studies, which did not allow us to formally summarize the data or perform a meta-analysis. We also combined studies with different definitions of MEs or administration errors. Finally, we included studies that did not mention whether they used the technique of disguised direct observation, whether the observers were previously trained, or whether the observations were validated.

This review shows the need for further studies in other countries to build a more comprehensive outlook on medication administration errors. Further studies using the disguised direct observation technique are required to achieve a more accurate estimate of the nature of medication administration errors. Another issue that needs more detail is the evaluation of the severity of the errors as none of the studies, even those that proposed to do so, carried out this type of analysis, which is of fundamental importance for good risk management.

## 5. Conclusions

The administration error rate is high in Latin America even when time errors are excluded. The primary errors in medication administration described in the studies were time, dose, omission, and administration route. The pharmacological groups most involved in medication administration errors were anti-infectives, central nervous system agents, blood and forming organs, cardiovascular system, digestive system, metabolism, and respiratory system. However, no study has yet evaluated the severity of medication administration errors. Future research using a broader disguised direct observation technique is required to obtain a more accurate estimate of the nature and severity of medication administration errors in Latin America.

## Supporting information

S1 Appendix Search strategies used in the literature review. (DOCX)

S2 Appendix PRISMA checklist. (DOCX)

S3 Appendix Quality study evaluation. (DOCX)

S1 Annex Medication error definitions. (DOCX)
