# Supplementary material for: Drug administration errors in Latin America: A systematic review
Source: PLoS One. 2022 Aug 4;17(8):e0272123. doi: 10.1371/journal.pone.0272123 (PMC9352042; doi:10.1371/journal.pone.0272123)
Supplement: S1 Appendix — (DOCX) [file pone.0272123.s001.docx]

**S1 APPENDIX. Search strategies used in the literature review**

| **INFORMATION SOURCE** | **SEARCH STRATEGY** | **RESULTS** |
| --- | --- | --- |
| **PubMed** | ("medication error$" OR "administration error$" OR "medication preparation" OR "omission error$" OR "medication handling") AND hospital$ AND ("latin america" OR argentina OR bolivia OR brazil OR chile OR colombia OR "costa rica" OR cuba OR "el salvador" OR ecuador OR guatemala OR haiti OR honduras OR mexico OR nicaragua OR panama OR paraguay OR peru OR "puerto rico" OR "dominican republic" OR uruguay OR venezuela)' | 26 |
|  | ("medication error" OR "medication errors" OR "administration error" OR "administration errors" OR "medication preparation" OR "omission error" OR "omission errors" OR "medication handling") AND hospital$ AND ("latin america" OR argentina OR bolivia OR brazil OR chile OR colombia OR "costa rica" OR cuba OR "el salvador" OR ecuador OR guatemala OR haiti OR honduras OR mexico OR nicaragua OR panama OR paraguay OR peru OR "puerto rico" OR "dominican republic" OR uruguay OR venezuela) | 196 |
| **BIREME** | (“error de medicacion” OR “errores de medicacion” OR “error de administración” OR “errores de administración”) AND (hospital OR hospitales) AND ("América Latina" OR Argentina OR Bolivia OR Brasil OR Chile OR Colombia OR "Costa Rica" OR Cuba OR "El Salvador" OR Ecuador OR Guatemala OR Haití OR Honduras OR México OR Nicaragua OR panama OR paraguay OR perú OR "puerto rico" OR "república dominicana" OR uruguay OR venezuela) | 295 |
|  | (“erro de medicação” OR “erros de medicação” OR “erro de administração” OR “erros de administração”) AND (hospital OR hospitais) AND ("América Latina" OR Argentina OR Bolivia OR Brasil OR Chile OR Colombia OR "Costa Rica" OR Cuba OR "El Salvador" OR Equador OR Guatemala OR Haiti OR Honduras OR México OR Nicaragua OR Panama OR paraguai OR peru OR "porto rico" OR "república dominicana" OR uruguai OR venezuela) | 138 |
|  | ('medication error' OR 'medication errors' OR 'administration error' OR 'administration errors') AND (hospital OR hospitals) ('latin america' OR argentina OR bolivia OR brazil OR chile OR colombia OR 'costa rica' OR cuba OR 'el salvador' OR ecuador OR guatemala OR haiti OR honduras OR mexico OR nicaragua OR panama OR paraguay OR peru OR 'puerto rico' OR 'dominican republic' OR uruguay OR venezuela) | 11 |
| **SCIELO** | (“erro de medicação” OR “erros de medicação” OR “erro de administração” OR “erros de administração”) AND (hospital OR hospitais) | 10 |
|  | (“error de medicacion” OR “errores de medicacion” OR “error de administración” OR “errores de administración”) AND (hospital OR hospitales) | 43 |
| **SCOPUS** | ("medication error" OR "medication errors" OR "administration error" OR "administration errors" OR "medication preparation" OR "omission error" OR "omission errors" OR "medication handling") AND hospital AND ("latin america" OR argentina OR bolivia OR brazil OR chile OR colombia OR "costa rica" OR cuba OR "el salvador" OR ecuador OR guatemala OR haiti OR honduras OR mexico ) | 175 |
|  | ("medication error" OR "medication errors" OR "administration error" OR "administration errors" OR "medication preparation" OR "omission error" OR "omission errors" OR "medication handling") AND hospital AND (nicaragua OR panama OR paraguay OR peru OR "puerto rico" OR "dominican republic" OR uruguay OR Venezuela) | 2 |
| **CINAHL** | ("medication error" OR "medication errors" OR "administration error" OR "administration errors" OR "medication preparation" OR "omission error" OR "omission errors" OR "medication handling") AND hospital AND ("latin america" OR argentina OR bolivia OR brazil OR chile OR colombia OR "costa rica" OR cuba OR "el salvador" OR ecuador OR guatemala OR haiti OR honduras OR mexico OR nicaragua OR panama OR paraguay OR peru OR "puerto rico" OR "dominican republic" OR uruguay OR venezuela) | 186 |
| **EMBASE** | ('medication error'/exp OR 'medication error*' OR 'administration error*' OR 'medication preparation' OR 'omission error' OR 'medication handling') AND hospital* AND ('latin america' OR 'argentina' OR 'bolivia' OR 'brazil' OR 'chile' OR 'colombia' OR 'costa rica' OR 'cuba' OR 'el salvador' OR 'ecuador' OR 'guatemala' OR 'honduras' OR 'mexico' OR 'nicaragua' OR 'panama' OR 'paraguay' OR 'peru' OR 'puerto rico' OR 'dominican republic' OR 'uruguay' OR 'venezuela') | 472 |
| **LATINDEX** | erro AND medicacao | 72 |
|  | error AND medicacion | 71 |
|  | erro AND administracao | 10 |
