# Supplementary material for: Drug administration errors in Latin America: A systematic review
Source: PLoS One. 2022 Aug 4;17(8):e0272123. doi: 10.1371/journal.pone.0272123 (PMC9352042; doi:10.1371/journal.pone.0272123)
Supplement: S3 Appendix — (DOCX) [file pone.0272123.s003.docx]

| **Section and Topic** | **Item #** | **Checklist item** | **Location where item**  **is reported** |
| --- | --- | --- | --- |
| **RESULTS** | | |  |
| Study selection | 16a | Describe the results of the search and selection process, from the number of records identified in the search to the number of studies included in the review, ideally using a flow diagram. | 6; fig 1 |
|  | 16b | Cite studies that might appear to meet the inclusion criteria, but which were excluded, and explain why they were excluded. | Fig. 1 |
| Study characteristics | 17 | Cite each included study and present its characteristics. | Table 1 |
| Risk of bias in studies | 18 | Present assessments of risk of bias for each included study. | Table 2 |
| Results of individual studies | 19 | For all outcomes, present, for each study: (a) summary statistics for each group (where appropriate) and (b) an effect estimate and its precision (e.g. confidence/credible interval), ideally using structured tables or plots. | Table 1 |
| Results of syntheses | 20a | For each synthesis, briefly summarise the characteristics and risk of bias among contributing studies. | 7 to 11 |
|  | 20b | Present results of all statistical syntheses conducted. If meta-analysis was done, present for each the summary estimate and its precision (e.g. confidence/credible interval) and measures of statistical heterogeneity. If comparing groups, describe the direction of the effect. | 7 to 11 |
|  | 20c | Present results of all investigations of possible causes of heterogeneity among study results. | 11 to 12 |
|  | 20d | Present results of all sensitivity analyses conducted to assess the robustness of the synthesized results. | - |
| Reporting biases | 21 | Present assessments of risk of bias due to missing results (arising from reporting biases) for each synthesis assessed. | 11 to 12 |
| Certainty of evidence | 22 | Present assessments of certainty (or confidence) in the body of evidence for each outcome assessed. | - |
| **DISCUSSION** | | |  |
| Discussion | 23a | Provide a general interpretation of the results in the context of other evidence. | 11 to 12 |
|  | 23b | Discuss any limitations of the evidence included in the review. | 10 to 12 |
|  | 23c | Discuss any limitations of the review processes used. | 11 to 12 |
|  | 23d | Discuss implications of the results for practice, policy, and future research. | 10 to 12 |
| **OTHER INFORMATION** | | |  |
| Registration and protocol | 24a | Provide registration information for the review, including register name and registration number, or state that the review was not registered. | - |
|  | 24b | Indicate where the review protocol can be accessed, or state that a protocol was not prepared. | - |
|  | 24c | Describe and explain any amendments to information provided at registration or in the protocol. | - |
| Support | 25 | Describe sources of financial or non-financial support for the review, and the role of the funders or sponsors in the review. | 14 |
| Competing interests | 26 | Declare any competing interests of review authors. | 14 |
| Availability of data, code and other materials | 27 | Report which of the following are publicly available and where they can be found: template data collection forms; data extracted from included studies; data used for all analyses; analytic code; any other materials used in the review. | - |

*From:* Page MJ, McKenzie JE, Bossuyt PM, Boutron I, Hoffmann TC, Mulrow CD, et al. The PRISMA 2020 statement: an updated guideline for reporting systematic reviews. BMJ 2021;372:n71. doi: 10.1136/bmj.n71

For more information, visit: <http://www.prisma-statement.org/>

**S3 APPENDIX. Quality study evaluation.**

|  | **ANSELMI, 2007** | **COSTA, 2006** | **OPTIZ, 2006** | **DE BORTOLI, 2010** | **CASSIANI, 2010** | **VOLPE, 2014** | | **MENDES, 2018** |
| --- | --- | --- | --- | --- | --- | --- | --- | --- |
| Were the criteria for inclusion in the sample clearly defined? | YES | YES | YES | YES | UNCLEAR | YES | NO | |
| Were the study subjects and the setting described in detail? | YES | NO | YES | NO | NO | YES | NO | |
| Was the exposure measured in a valid and reliable way? | YES | YES | YES | YES | UNCLEAR | YES | UNCLEAR | |
| Were objective, standard criteria used for measurement of the condition? | YES | YES | YES | UNCLEAR | UNCLEAR | UNCLEAR | UNCLEAR | |
| Were confounding factors identified? | YES | YES | YES | YES | YES | YES | YES | |
| Were strategies to deal with confounding factors stated? | YES | NO | NO | NO | NO | YES | UNCLEAR | |
| Were the outcomes measured in a valid and reliable way? | YES | YES | YES | UNCLEAR | YES | UNCLEAR | UNCLEAR | |
| Was appropriate statistical analysis used? | YES | YES | UNCLEAR | UNCLEAR | UNCLEAR | YES | YES | |
|  |  |  |  |  |  |  |  | |
| Critérios atendidos | 100% | 75% | 75% | 38% | 25% | 75% | 25% | |
|  |  |  |  |  |  |  |  | |
| **Overall bias risk** | **Low** | **Moderate** | **Moderate** | **High** | **High** | **Moderate** | **High** | |
|  |  |  |  |  |  |  |  | |

**JBI checklist for analytical cross-sectional studies.**

Bias risk assessment: Low risk: 76 to 100% of criteria; Moderate risk: 51 to 75% of criteria; High risk: 0 to 50% of criteria.

**Newcastle-Ottawa Quality Assessment Form for Cohort Studies.**

|  | **ROMERO, 2013** | **SMITH, 2014** | **REIS, 2010** |
| --- | --- | --- | --- |
| **SELECTION** |  |  |  |
| Representativeness of the exposed cohort | Truly representative (one star) | Truly representative (one star) | No description of the derivation of the cohort |
| Selection of the non-exposed cohort | Drawn from a different source | No description of the derivation of the non exposed cohort | No description of the derivation of the non exposed cohort |
| Ascertainment of exposure | Structured interview (one star) | Structured interview (one star) | Structured interview (one star) |
| Demonstration that outcome of interest was not present at start of study | No | No | No |
| **COMPARABILITY** |  |  |  |
| Comparability of cohorts on the basis of the design or analysis controlled for confounders | Study controls for other factors (one star) | Study controls for other factors (one star) | Study controls for other factors (one star) |
| **OUTCOME** |  |  |  |
| Assessment of outcome | Self report | Self report | Self report |
| Was follow-up long enough for outcomes to occur | Yes (one star) | Yes (one star) | Yes (one star) |
| Adequacy of follow-up of cohorts | No statement | No statement | No statement |
| **Overall bias risk** | **Fair quality** | **Fair quality** | **Poor quality** |

Good quality: 3 or 4 stars in selection domain AND 1 or 2 stars in comparability domain AND 2 or 3 stars in outcome/exposure domain

Fair quality: 2 stars in selection domain AND 1 or 2 stars in comparability domain AND 2 or 3 stars in outcome/exposure domain

Poor quality: 0 or 1 star in selection domain OR 0 stars in comparability domain OR 0 or 1 stars in outcome/exposure domain
