# Supplementary material for: Drug administration errors in Latin America: A systematic review
Source: PLoS One. 2022 Aug 4;17(8):e0272123. doi: 10.1371/journal.pone.0272123 (PMC9352042; doi:10.1371/journal.pone.0272123)
Supplement: S1 Annex — (DOCX) [file pone.0272123.s004.docx]

**S1 ANNEX . Medication error definitions.**

| **Author** | **Definition** |
| --- | --- |
| BARKER^25^ | "A medication error is generally defined as a deviation from the physician's medication order as written on the patient's chart." |
| ASHP^23^ | "Episodes in drug misadventuring that should be preventable through effective systems controls involving pharmacists, physicians and other prescribers, nurses, risk management personnel, legal counsel, administrators, patients, and others in the organizational setting, as well as regulatory agencies and the pharmaceutical industry" |
| NCC MERP^24^ | "Any preventable event that may cause or lead to inappropriate medication use or patient harm while the medication is in the control of the health care professional, patient or consumer." |
| FERNER; ARONSON^37^ | "A failure in the treatment process that leads to or has the potential to lead to harm to the patient" |
